# Detection of Invisible Damages in ‘Rojo Brillante’ Persimmon Fruit at Different Stages Using Hyperspectral Imaging and Chemometrics

**DOI:** 10.3390/foods10092170

**Published:** 2021-09-13

**Authors:** Sandra Munera, Alejandro Rodríguez-Ortega, Nuria Aleixos, Sergio Cubero, Juan Gómez-Sanchis, José Blasco

**Affiliations:** 1 Centro de Agroingeniería, Instituto Valenciano de Investigaciones Agrarias (IVIA), Carretera CV-315, Km 10.7, 46113 Moncada, Spain; munera_san@gva.es (S.M.); cubero_ser@gva.es (S.C.); 2 Departamento de Ingeniería Gráfica, Universitat Politècnica de València, Camino de Vera, s/n, 46022 Valencia, Spain; alrodor@alumni.upv.es (A.R.-O.); naleixos@dig.upv.es (N.A.); 3 Intelligent Data Analysis Laboratory, Department of Electronic Engineering, ETSE (Engineering School), Universitat de València (UV), Av. Universitat, s/n, Bujassot, 46100 Valencia, Spain; Juan.Gomez-Sanchis@uv.es

**Keywords:** *Diospyros kaki*, fruit quality, browning, nondestructive, chemometrics, computer vision

## Abstract

The main cause of flesh browning in ‘Rojo Brillante’ persimmon fruit is mechanical damage caused during harvesting and packing. Innovation and research on nondestructive techniques to detect this phenomenon in the packing lines are necessary because this type of alteration is often only seen when the final consumer peels the fruit. In this work, we have studied the application of hyperspectral imaging in the range of 450–1040 nm to detect mechanical damage without any external symptoms. The fruit was damaged in a controlled manner. Later, images were acquired before and at 0, 1, 2 and 3 days after damage induction. First, the spectral data captured from the images were analysed through an algorithm based on principal component analysis (PCA). The aim was to automatically separate intact and damaged fruit, and to detect the damage in the PC images when present. With this algorithm, 90.0% of intact fruit and 90.8% of damaged fruit were correctly detected. A model based on partial least squares—discriminant analysis (PLS-DA), was later calibrated using the mean spectrum of the pixels detected as damaged, to determine the moment when the fruit was damaged. The model differentiated fruit corresponding correctly to 0, 1, 2 and 3 days after damage induction, achieving a total accuracy of 99.4%.

## 1. Introduction

‘Rojo Brillante’ is the most cultivated persimmon fruit in Spain and the Mediterranean region. Consumers appreciate this fruit for its good appearance, large size and flavour, and the absence of seeds [1]. In Spain, this cultivar obtained the Protected Designation of Origin ‘Kaki Ribera del Xúquer’ in 1998. In recent years, the commercial success experienced by this astringent cultivar has been promoted by a deastringency treatment, based on exposure to high CO_2_ concentrations [2], which also preserves the firm and crispy texture of the fruit.

One of the leading postharvest problems of persimmon ‘Rojo Brillante’ is currently the incidence of flesh browning [3]. This browning begins in the most superficial area of the fruit pulp and becomes deeper over time [4]. Besada et al. [5] found that this disorder is caused mainly by the fruit being hit and suffering mechanical damage during packing operations. The solubility of tannins (responsible for the astringency) seems to be a crucial factor in persimmon browning as deastringed fruit is more susceptible to impact-induced browning. In contrast, astringent fruit is less sensitive to these packing operations [3]. This problem could be reduced by using manual handling or designing packing lines that are better adapted to this fruit. 

However, when this damage occurs, it is necessary to apply inspection techniques that can detect it early in order to prevent damaged fruit from reaching the consumer [6]. The problem faced by industry is that this alteration can develop without any visible symptoms, which prevents manual or standard inspection systems from detecting the damage and causes the consumer to find it when peeling the fruit, thus causing it to be rejected.

This technique has demonstrated the ability to detect mechanical damage in fruit and vegetables such as citrus fruit [7], apples [8,9], peaches [10,11], pears [12], mangos [13], blueberries [14], loquats [15] or potatoes [16,17]. Regarding persimmon fruit, hyperspectral imaging has been studied as a mean to assess the internal quality such as firmness, soluble solids content and maturity [1,18] or astringency [19]. However, this technology has not yet been applied to detect internal damage in this fruit.

One of the main drawbacks of this technology is the massive amount of data generated that need to be processed quickly using multivariate analysis or chemometric methods. Principal component analysis (PCA) is a method commonly used in hyperspectral image processing both to reduce the data dimensionality and to obtain an overview of important information, such as damage or defects [11,16,20]. Conversely, if classification is needed, partial least squares—discriminant analysis (PLS-DA) is another popular method chosen by numerous authors to create predictive models in the discrimination of damages or defects [10,17,21]. Therefore, this work aims to investigate the potential of hyperspectral imaging in combination with chemometric methods to evaluate the internal quality of the ‘Rojo Brillante’ persimmon fruit. We present a novel approach capable of detecting invisible mechanical damage using a PCA-based algorithm and, once a damage is detected, determining when it occurred using PLS-DA.

## 2. Materials and Methods

### 2.1. Fruit Samples and Experimental Design

In this study, a total of 65 persimmon fruit (*Diospyros kaki* cv. ‘Rojo Brillante’) of a similar size and apparently free of any visible bruising or defects were analysed. The fruit was harvested in L’Alcudia (Valencia, Spain) at commercial maturity. It was then exposed to CO_2_ treatment in closed containers (air containing 95% CO_2_ at 20 °C and relative humidity of 90%) for 24 h to remove the astringency [2].

**Figure 1:**
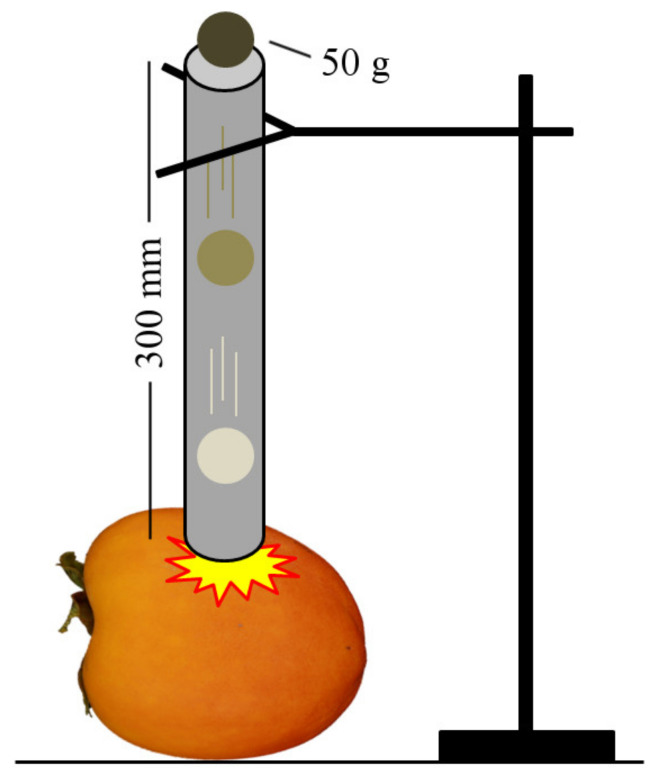
Mechanical damage device.

An impact device was used to induce internal damage artificially (Figure 1). This instrument consisted of a vertical polyvinyl chloride (PVC) tube resting on the fruit. A 50 g ball was dropped from a height of 30 mm above the surface of the fruit. The height and the weight of the ball were calculated in such a way as to ensure it would damage the flesh without causing any external symptoms. Since no previous studies that induce artificial damage in persimmon were found, it was decided, based on our extensive experience with this fruit [1,2,3,4,5,19], to cause an impact with an energy of 0.2 J. The impact was induced in the two faces of the equatorial area of the fruit.

**Figure 2:**
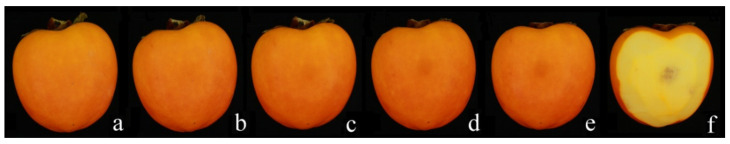
Appearance of intact (**a**) and damaged fruit after 0 (**b**), 1 (**c**), 2 (**d**) and 3 days (**e**,**f**).

Hyperspectral and RGB images of the same fruit were acquired before hitting the (intact) fruit, just after damage induction (0 days), and after 1, 2 and 3 days (Figure 2), which led to the capture of a total of 650 hyperspectral images. Finally, the fruit was peeled to visualise the browning on the flesh (Figure 2f). The fruit was stored under constant conditions between each image acquisition. The storage temperature was 20 °C and the RH was 90%.

### 2.2. Image Acquisition

The hyperspectral imaging system used in this work was previously described in Munera et al. [15]. It consisted of an industrial camera (CoolSNAP ES, Photometrics, Tucson, AZ, USA), coupled to two liquid-crystal tuneable filters (Varispec VIS-07 and NIR-07, Cambridge Research & Instrumentation, Inc., Hopkinton, MA, USA). The camera was configured to acquire images with a size of 1392 × 1040 pixels and a spatial resolution of 0.14 mm/pixel at 60 different wavelengths every 10 nm, in the operating spectral range of 450–1040 nm. To optimise the dynamic range of the camera and prevent saturated regions, a calibration of the integration time of each band was performed by capturing the averaged grey level of a white reference target (Spectralon 99%, Labsphere, Inc., North Sutton, NH, USA) corresponding to 90% of the dynamic range of the camera.

The scene was illuminated by indirect light from twelve halogen spotlights (37 W) (Eurostar IR Halogen MR16. Ushio America, Inc., Tokyo, Japan) powered by direct current (12 V) and arranged equidistant from each other inside a hemispherical aluminium diffuser. The inner surface of the aluminium diffuser was painted white with a rough texture to maximise its reflectivity, the rough texture being applied to minimise directional reflections, which could cause bright spots, the result being highly homogeneous light.

Hyperspectral images of the fruit were obtained with the induced damage facing the camera. White and dark reference images were also acquired using the white calibration target (99% reflectance) and covering the lens with the cap, respectively. A total of 650 images were obtained, of which 130 corresponded to each state: sound fruit before causing the damage, just after the damage (0 d), and 1, 2, and 3 days after the test.

RGB images were also acquired to visually evaluate the evolution of the damage over time (Figure 2). The RGB acquisition system consisted of a digital camera (EOS 550D, Canon Inc., Tokyo, Japan) arranged inside a square inspection chamber with an illumination system made up of eight BIOLUX 18W/965 (Osram GmbH, Munich, Germany) fluorescent tubes with a colour temperature of 6500 K. The angle between the axis of the lens and the sources of illumination was set to 45°. Additionally, polarising filters were placed in front of the lamps and camera lenses to eliminate specular bright spots that could alter the actual colour.

### 2.3. Image Processing and Analysis

#### 2.3.1. Fruit Segmentation and Damage Detection

Hyperspectral images were processed and analysed using MATLAB R2020b (The MathWorks, Inc., Natick, MA, USA). Image processing started with the correction of the relative reflectance using the white and a dark reference by means of Equation (1) [22]:

(1) $$\rho_{xy}\left(x,y,\lambda \right)=\frac{R^{abs}}{R_{white}^{abs}}=\rho_{ref}\left(\lambda \right)\frac{R\left(x,y,\lambda \right)-R_{black}\left(x,y,\lambda \right)}{R_{white}\left(x,y,\lambda \right)-R_{black}\left(x,y,\lambda \right)}$$

where *ρ_ref_*(*λ*) is the reflectance of the white calibration target (99%), *R*(*x*, *y*, *λ*) is the reflectance of the fruit captured by the sensor of the camera, *R_white_*(*x*, *y*, *λ*) is the reflectance of the white reference target, and *R_black_*(*x*, *y*, *λ*) is the reflectance captured while avoiding any light source in order to quantify the electronic noise of the sensor.

After that, a nonlocal meet global filter [23] was applied to reduce the instrumental noise and thus improve the signal-to-noise ratio of the hyperspectral images [24]. Automatic detection of the damages was done using PCA in two steps. First, the background and the leaves of the calyx were removed, leaving only the fruit. Second, the damages were automatically detected in the fruit. PCA condenses the information from a dataset by applying a transformation to create new variables as uncorrelated and linear combinations of the original variables [25]. This method sorts these new variables, or principal components (PC), the first ones explaining the most variability and thus enhance the main features present in the hyperspectral image [26]. A PCA-based model was therefore performed in this work to transform the denoised hyperspectral images into PC images.

**Figure 3:**
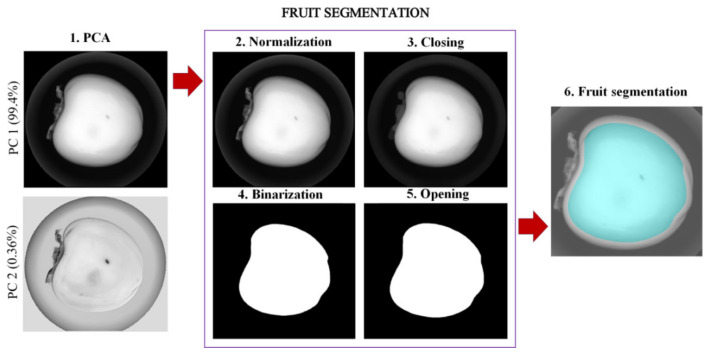
Steps performed for background, calyx and fruit segmentation.

The steps to remove the background and the calyx from the images are shown in Figure 3. For this process, some authors used images at given wavelengths where the contrast between the background and the foreground is maximum and then segmented the fruit by thresholding [20,27]. However, in this case, thresholding did not perform well as the leaves of the calyx were not well segmented in all cases, so a more sophisticated and unsupervised method, such as PCA, was used. The PC1 image explains most of the variance in the data (99.42%), showing the information on the physical properties of the fruit, enhancing the contrast of the different elements of the acquired hyperspectral image, such as the background, the calyx leaves, and the fruit. Alternatively, the PC2 image (0.36%) included information about illumination distribution [26]. The PC1 image was therefore chosen to separate the fruit from the background and the calyx. This image was then normalised following Equation (2) so that each pixel had a value between 0 and 1.

(2) $$PC1_{norm}=\frac{PC1-min\left(PC1\right)}{max\left(PC1\right)-min\left(PC2\right)}$$

A closing morphological transform was applied to remove noise inside each object without affecting the overall shape [28,29]. To obtain the fruit segmentation mask, the closing image was then binarised using the Otsu method [30] which is an adaptative segmentation method. Finally, a morphological opening operation was applied to remove the possible presence of small or other objects near the boundary caused by the spherical shape of the fruit.

**Figure 4:**
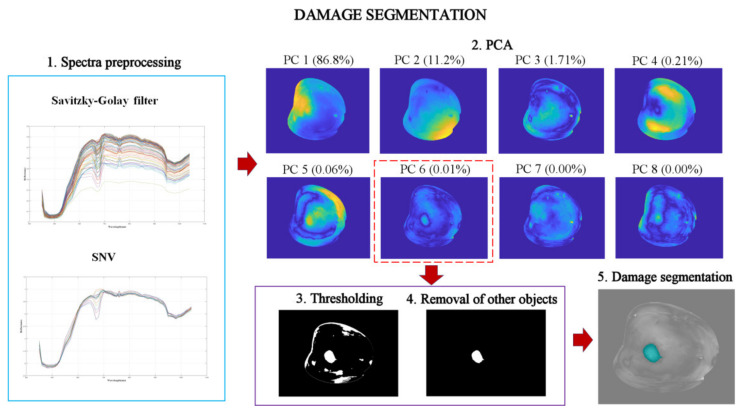
Steps performed for damage detection.

Once the region of interest (fruit only) had been selected, the next step was to detect the damage. The damage segmentation pipeline is depicted in Figure 4. To build the model for detecting damaged fruit, two images from each day after damage induction (days 0 to 3) were randomly selected. First, the spectra were preprocessed using a Savitzky–Golay filter, with a five-point frame length and a second-order polynomial to smooth the spectral signal while preserving its shape, and thus reduce the physiological noise. After that, standard normal variate (SNV) was applied to minimise the effect of light scattering [31]. A second PCA was then carried out on the images after removing the background and the calyx. The first eight PC images (PC1-PC8), containing the cumulative explained variance of 99.99%, were chosen to find the PC images that were more suitable for damage segmentation. Even though the first PC images explained most of the data variance, the damage contour was better delimited in the PC6 image than in the others (Figure 4). According to other similar studies, the most suitable PC image to segment damage is usually chosen visually [20,25]. Therefore, the PC6 image was selected. Again, an adaptative thresholding based on the Otsu method was applied in this PC6 image to discriminate damaged pixels from the intact pixels. After that, some pixels near the edge could be misclassified due to a lower illumination caused by the curvature of the fruit (Figure 4). To avoid these expected errors, the area and circularity of each object in the binary image were calculated to remove long and narrow structures compatible with errors close to the edge [20]. This algorithm was applied to a total of 642 images of which 130 corresponded to intact fruit and 512 images to damaged fruit (128 images for each day after damage induction).

#### 2.3.2. Damage Discrimination

Once damages were detected, the discrimination of the time in which they were caused was carried out using a PLS-DA model. Hence, the model was carried out only for the fruits detected as damaged, using the raw mean spectra of the region detected as damaged in the previous step. PLS-DA is based on PLS regression, which searches for a linear regression model of latent variables by projecting prediction variables X and response variables Y into a new latent space where the covariance between these latent variables is maximised. The aim was to find the latent multidimensional direction in the data space that explained the direction of the maximum multidimensional covariance in the reference properties space. When the Y variable is categorical, as in this case, PLS-DA is performed to sharpen the separation between groups of observations by maximising the covariance between, for example, the spectral data and the days after damage induction, such that a maximum separation among these classes is obtained [25]. 

In this case, the spectra were distributed in a matrix with 60 columns, each corresponding to the reflectance value of each wavelength (450–1050 nm), while the rows represented the mean spectra of the damage region of each damaged fruit. The mean spectra were labelled as belonging to any day after damage induction (0 d, 1 d, 2 d and 3 d). To reduce the variability among samples due to light scattering [31], the raw mean spectra were preprocessed using the Savitzky-Golay derivative (three-point smoothing window, second-order polynomial) and SNV. Mean centring was applied to normalise the whole spectrum. The spectra were randomly partitioned into two sets: two-thirds of the samples correctly detected as damaged by the previous PCA algorithm were used to calibrate the model (training set) and cross-validation (CV), while the remaining third of the damaged samples was used as test set. The optimal number of latent variables (LV) was selected using a 10-fold CV on the training set.

The results of the PLS-DA model were shown in a confusion matrix, expressed as a percentage of correct classification for the calibration, CV and prediction sets. Finally, these results of the prediction were visualised on the image of the surface of the fruit.

## 3. Results and Discussion

### 3.1. Spectral Analysis

**Figure 5:**
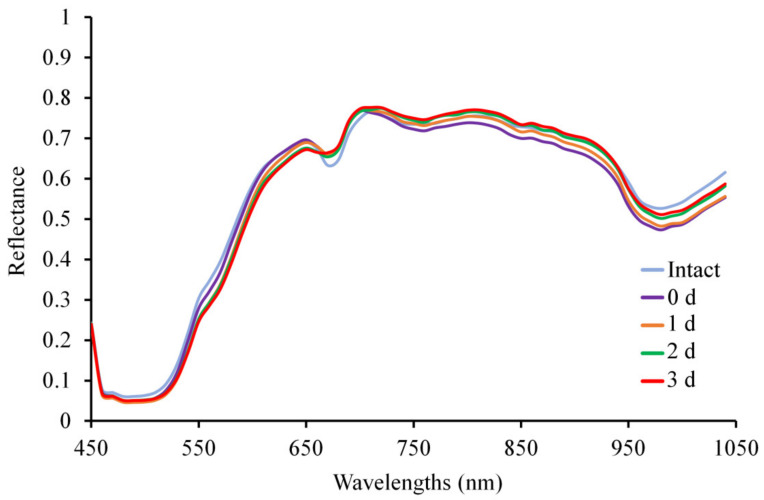
Raw mean spectra of intact and damaged areas over time.

The raw mean spectra of intact and damaged regions of interest captured at the centre of the fruit at different days are presented in Figure 5. In the VIS region, the reflectance of the intact fruit spectrum was higher than the damaged region spectra from 450 to 660 nm, decreasing according to the time after damage induction and the opposite occurred at 670 nm. This could be due to the appearance of browning in the flesh of damaged fruit, which can change the colour of the skin in this area [3]. At a molecular level, these changes over time can be associated with the degradation of carotenoids and chlorophylls [27,32]. The intact fruit spectrum was also higher in the NIR region than the damaged region, especially in the valley around 970 nm associated with the water absorption peak [33]. However, the evolution of the damage spectra over time was different to that of the VIS region. The spectrum at 0 d presented the lowest reflectance (or highest absorbance) of water, and the spectrum at 3 d showed the contrary. These changes are due to the degradation of the parenchyma due to the blow with the ball [3]. An osmotic movement of water is thus produced from the flesh to the skin leaving free water in the tissues (0 d–1 d) and, as time passes, some degree of dryness appears (2 d–3 d) [17,32]. In the case of the intact fruit spectrum, since the tissues were not damaged and there was no water outside them, its reflectance was the highest.

### 3.2. Damage Segmentation

**Figure 6:**
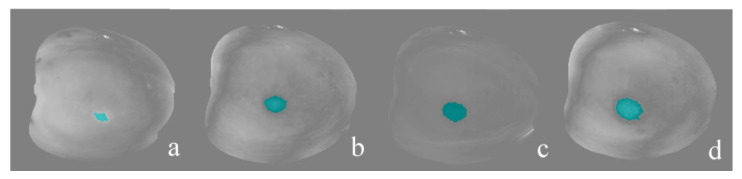
Segmentation of disguised damage (in blue) 0 (**a**), 1 (**b**), 2 (**c**) and 3 days (**d**) after damage induction using the PCA-based algorithm in the PC6 image.

The results of the damage detection using PCA are presented in Figure 6 and Table 1. Figure 6 shows the segmentation of the damage regions in the PC6 image in greyscale of fruit each day after damage induction. The pixels identified as damaged were coloured in blue by the segmentation algorithm. Although RGB images of the peeled fruit were not acquired on each day of analysis (only on the last day), in this figure, it is possible to observe an increase in the area of the damage over time. This fact was also detected by El Masry et al. [27] when attempting to detect damages in apples after 1 h and three days.

As for the accuracy of detection of intact and damaged fruits (Table 1), 90.0% of intact fruits were correctly identified because the algorithm did not detect any damage pixel in the image, and therefore no region was segmented. Regarding the damaged fruit, the 77.3%, 90.6%, 96.1% and 99.2% were correctly identified for each day after damage induction. The accuracy of classification of both intact and damaged fruit was 90.8%.

These results are consistent with Novillo et al. [3], who observed that browning in the flesh during storage at 15 °C occurred around 12 h after the damage is caused during packing. In that time, only 10% of the fruit was slightly browned. From 18 h on, all fruit manifested browning. Furthermore, in a microstructure study, they found that browned flesh areas showed a fairly degraded parenchyma. These areas were characterised by the presence of tannic cells in which tannins precipitated after the deastringency treatment. While no coloured cells were encountered in intact flesh, these cells were intensely red-brown coloured in browned flesh. For this reason, the lower precision at 0 d was because the images were acquired just after damage induction when the internal browning had not yet appeared. The increased accuracy of the rest of the fruits from day 1 was due to this gradual appearance of browning [3] and the increase in the size of the damaged area, which facilitate the identification of damaged pixels in the fruit, as shown in Figure 6.

As commented earlier, numerous studies have used PCA and different morphological operations to segment damage in fruit or vegetables with high accuracy. For example, Li et al. [11] used PC images and a watershed segmentation to segment damaged and intact peaches, obtaining accuracy rates of 96.5% and 97.5%, respectively. Ji et al. [16] identified damage in potatoes with different methods such as future extraction, obtaining a total accuracy of 93.8% when PCA was used. Gowen et al. [17] segmented bruises in mushrooms with an accuracy of 89.5% for intact mushrooms and 87.3% for mushrooms bruised under different conditions. These results were similar to those obtained in this study. However, in some cases, the bruises or damage were easily visible in their respective RGB images. In the present work, the difficulty to segment the damaged regions, especially just after damage induction (0 d), was that the browning of the flesh usually appears, at least, after 12 h. Although the damage evolved, it could not be easily detected by the naked eye at least three days after damaging the fruit, especially during intensive inspection labour.

### 3.3. Damage Discrimination

Since the damaged area was delimited in the previous segmentation step, the mean spectrum of these areas was used instead of individual pixel spectra. The aim was to obtain more stable classification models and to avoid classification by pixels which can be easily misassigned to one of the other three classes [34,35].

As a calibration set, 310 fruits (two-thirds of the samples detected as damaged) were selected randomly, while the remaining third (155 fruits) were used as the independent test set. The model was performed using 15 LVs since the error rate was the lowest using this number of LVs in the CV. The results indicated that 100% of samples from each day after damage induction were correctly discriminated (Table 2). For CV, the accuracy decreased slightly on 1 d and 2 d, to 97.5% and 98.8%, respectively. Thus, total accuracy decreased to 99.1%.

The results for the test set were like those obtained in the calibration, showing an accuracy of 97.4% for 1 d and 100% for the rest of the days after damage induction, achieving a total accuracy of 99.4%. These results are like those obtained in the discrimination of damages in peaches, apples, or mangoes [8,9,10,13]. These results suggest that hyperspectral imaging could be applied in persimmon fruit with great precision to determine when the fruit was damaged.

**Figure 7:**
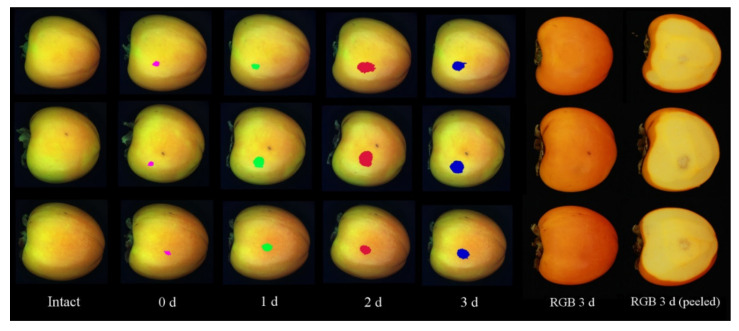
Examples of the segmentation and discrimination of the damage over time in the hyperspectral images (the first five columns) of three fruits using the PCA-based algorithm and the PLS-DA model. The two columns on the right are the RGB images of these fruits captured on the last day, before and after peeling.

Only for visual testing, three fruits belonging to the test set were used to obtain a graphical view of the segmentation and the discrimination of the damage (Figure 7). The damage region was coloured in magenta if the model assigned the mean value to 0 d, green to 1 d, red to 2 d and blue to 3 d after damage induction. In the intact fruit, no damage regions were identified. For comparison purposes, RGB images of these fruits before and after peeling them were also captured on 3 d.

## 4. Conclusions

In this work, the feasibility of hyperspectral imaging in the 450–1040 nm region to detect and discriminate disguised mechanical damage over time in ‘Rojo Brillante’ persimmon fruit has been demonstrated.

The detection of intact and damaged fruit was performed using an automatic algorithm based on PCA, achieving accuracy rates of 90.0% and 90.8%, respectively.

After damage detection, the mean spectrum of the damage regions obtained by the previous algorithm was used as input for a PLS-DA model. This model provided a discrimination accuracy of 100%, 97.4%, 100% and 100% for 0 d, 1 d, 2 d and 3 d, respectively. Furthermore, this prediction was put in a visual form on the images, thereby facilitating the observation of the detection and discrimination of disguised damage in the image of the fruit surface over time.

Since these results were achieved on controlled mechanical damages, they must be confirmed by passing and damaging the fruit on actual packing lines, to detect these possible hidden damages in an industrial setting. Once this has been achieved, selecting a set of optimal wavelengths is essential to speed up the process. Hyperspectral imaging may have potential as a tool for rapid and nondestructive damage detection and discrimination, which would prevent fruits that have been mechanically damaged before or during packing from reaching the final consumer.

## Figures and Tables

**Table 1:** Results of damage segmentation using the PCA-based algorithm.

Fruit	Intact	Damaged			
		0 Day	1 Day	2 Days	3 Days
Num. of samples	130	128	128	128	128
Correctly detected	117	99	116	123	127
	90.0%	77.3%	90.6%	96.1%	99.2%

**Table 2:** Results of discrimination of time after damage induction using PLS-DA model.

	0 Day	1 Day	2 Days	3 Days	Total (%)
Calibration					
0 day	64	0	0	0	100
1 day	0	79	0	0	100
2 days	0	0	83	0	100
3 days	0	0	0	84	100
Cross validation					
0 day	64	0	0	0	100
1 day	2	77	0	0	97.5
2 days	0	1	82	0	98.8
3 days	0	0	0	84	100
Test					
0 day	35	0	0	0	100
1 day	1	36	0	0	97.4
2 days	0	0	40	0	100
3 days	0	0	0	43	100

## Data Availability

Images used in the study can be found at http://www.cofilab.com.
